# Biochemical markers of bone turnover and their association with bone marrow lesions

**DOI:** 10.1186/ar2494

**Published:** 2008-08-29

**Authors:** David J Hunter, Michael LaValley, Jiang Li, Doug C Bauer, Michael Nevitt, Jeroen DeGroot, Robin Poole, David Eyre, Ali Guermazi, Daniel Gale, Saara Totterman, David T Felson

**Affiliations:** 1 Department of Epidemiology and Biostatistics, Boston University School of Medicine, Albany Street, Boston, Massachusetts 02118, USA; 2 New England Baptist Hospital, Parker Hill Avenue, Boston, Massachusetts 02120, USA; 3 University of California at San Francisco, Berry Street, San Francisco, California 94107, USA; 4 TNO Quality of Life, Business Unit Biomedical Research, Zernikedreef 9, 2333 CK Leiden, The Netherlands; 5 Joint Diseases Laboratory, McGill University, Cedar Avenue, Quebec, H3G 1A6, Canada; 6 Department of Orthopaedics and Sports Medicine, University of Washington, NE Pacific Street, Seattle, Washington 98195, USA; 7 Virtualscopics, Linden Oaks, Rochester, New York 14625, USA

## Abstract

**Introduction:**

Our objective was to determine whether markers of bone resorption and formation could serve as markers for the presence of bone marrow lesions (BMLs).

**Methods:**

We conducted an analysis of data from the Boston Osteoarthritis of the Knee Study (BOKS). Knee magnetic resonance images were scored for BMLs using a semiquantitative grading scheme. In addition, a subset of persons with BMLs underwent quantitative volume measurement of their BML, using a proprietary software method. Within the BOKS population, 80 people with BMLs and 80 without BMLs were selected for the purposes of this case-control study. Bone biomarkers assayed included type I collagen N-telopeptide (NTx) corrected for urinary creatinine, bone-specific alkaline phosphatase, and osteocalcin. The same methods were used and applied to a nested case-control sample from the Framingham study, in which BMD assessments allowed evaluation of this as a covariate. Logistic regression models were fit using BML as the outcome and biomarkers, age, sex, and body mass index as predictors. An receiver operating characteristic curve was generated for each model and the area under the curve assessed.

**Results:**

A total of 151 subjects from BOKS with knee OA were assessed. The mean (standard deviation) age was 67 (9) years and 60% were male. Sixty-nine per cent had maximum BML score above 0, and 48% had maximum BML score above 1. The only model that reached statistical significance used maximum score of BML above 0 as the outcome. Ln-NTx (Ln is the natural log) exhibited a significant association with BMLs, with the odds of a BML being present increasing by 1.4-fold (95% confidence interval = 1.0-fold to 2.0-fold) per 1 standard deviation increase in the LnNTx, and with a small partial R^2 ^of 3.05. We also evaluated 144 participants in the Framingham Osteoarthritis Study, whose mean age was 68 years and body mass index was 29 kg/m^2^, and of whom 40% were male. Of these participants 55% had a maximum BML score above 0. The relationship between NTx and maximum score of BML above 0 revealed a significant association, with an odds ratio fo 1.7 (95% confidence interval = 1.1 to 2.7) after adjusting for age, sex, and body mass index.

**Conclusions:**

Serum NTx was weakly associated with the presence of BMLs in both study samples. This relationship was not strong and we would not advocate the use of NTx as a marker of the presence of BMLs.

## Introduction

Research into the etiology and progression of knee osteoarthritis (OA) has focused on the destruction of articular cartilage. However, it is clear that knee OA is an organ-level failure of the joint and involves pathologic changes in articular cartilage as well as in subchondral bone [1].

The structural properties of subchondral bone may play a role in the degeneration of cartilage [2], and adjoining trabecular changes are considered necessary for the progression of OA [3]. In OA, bone has characteristic morphologic abnormalities, including altered joint congruency, bone marrow lesions (BMLs) [4], subchondral sclerosis, intraosseous cysts, and osteophytes.

BMLs are high-signal lesions in the medullary space that extende to subcortical bone, according to T_2_-weighted magnetic resonance imaging (MRI) [5]. The earliest investigations reflecting this disturbance were conducted using scintigraphy [6-8]. This work suggested that not only was the activity of the subchondral bone altered, but also this may determine the rate of loss of cartilage. In work from the Boston Osteoarthritis Knee Study (BOKS), BMLs on MRI were found to be strongly associated with the presence of pain in knee OA [4].

More importantly from the perspective of prognostic markers, the BOKS study findings suggested that BMLs are a powerful independent predictor of OA progression (joint space loss over time on fluoroscopically positioned x-ray) in persons with painful knee OA, and their presence identified most knees at risk for progression [9]. These findings replicate an earlier natural history study [7] in which bone scan lesions were found to powerfully predict radiographic progression in the knees.

On bone histology, areas of bone affected by BMLs exhibit reversal lines indicative of bone remodeling [10]. Small areas of bone necrosis are also present, as is scar. The extensive bone remodeling seen, and the correlation of MRI bone marrow lesions with positive bone scintigraphy (a tracer that is picked up by osteoblasts) both strongly suggest that bone turnover is markedly increased in these local areas.

Other evidence supporting a high rate of turnover in advancing OA emanates from studies suggesting that there is increased synthesis and degradation of the subchondral bone collagen in OA, as well as changes to post-translational modifications [11,12]: increased lysine hydroxylation, changes in the nature of the cross-links between the polypeptide chains, and consequent decreased mineralization.

Subjacent to the thickened cortical plate, studies have reported hypomineralization of trabecular bone [13-15]. This localized loss of bone mass is possibly linked to abnormal bone cell behavior in OA joints, reported as imbalances in bone resorption, formation, or both [16]. Recent studies have confirmed that increased bone resorption plays an integral role in the disease process, with increased levels of bone resorption markers reported in patients with radiographic evidence of knee OA, including type I collagen [17] and deoxypryidinoline [18]. In addition to bone being lost locally within the diseased joint, altered bone has been reported in OA patients at sites distant from weight-bearing joints [19], and low hip BMD appears to be correlated with OA knee progression [20,21]. One explanation for this change is that there is rapid turnover of subchondral bone in OA, and there is insufficient time for the bone to be adequately mineralized. It is not known whether these areas of abnormal bone are derived from areas that would generate BML findings on MRI.

We hypothesized that if bone marrow lesions are areas of accelerated bone turnover, then they should be accompanied by elevation in systemic levels of bone turnover markers. If our hypothesis is correct, namely that bone turnover markers are associated with BMLs and that – like BMLs – they predict cartilage loss, then this will provide important insights into the pathophysiology of OA and may provide a biomarker that, like the BML that it reflects, may potently predict cartilage loss.

## Materials and methods

We conducted a cross-sectional analysis of data from two sources: BOKS and the Framingham Osteoarthritis Study. Within each study 80 people with BMLs and 80 without BMLs were chosen for a nested case-control sample.

### BOKS Sample

We conducted a cross-sectional analysis of data from the Boston Osteoarthritis of the Knee Study (BOKS) [22] – a completed natural history study of knee OA. To be eligible for inclusion in the study, a person had to have knee pain, aching or stiffness on most days within the preceding month, and they had to have reported that a physician had told them that they had arthritis in the knee. If they met both of those criteria then they underwent radiography (weight-bearing fluoroscopic posteroanterior (PA), lateral, and skyline views), and if on any of these views they had a definite osteophyte in the symptomatic knee, then they were eligible for inclusion in the study. In addition, if they screened positive for another form of arthritis or were using medications that were appropriate for rheumatoid or other forms of arthritis, then they were excluded. Thus, all of those included in the study had primary clinical knee OA and met American College of Rheumatology criteria for this disorder [23].

Of 324 patients who entered the study, 86% completed a full comprehensive follow up at a later time point (either 15 or 30 months, or both). Comprehensive examinations consisted of an MRI of the more affected knee and a comprehensive set of radiographs, including a semiflexed fluoroscopically positioned PA radiograph using the method of Buckland-Wright [24].

Fasting blood and urine specimens were also obtained at baseline. Serum was frozen at -70°C and urine at -20°C. The specimens were stored at the Biomedical Research Institute in Rockville (MD, USA).

For grant funding reasons, we were funded to perform biomarkers on 160 of these participants. We conducted a nested study of persons within BOKS who had and did not have BMLs. After completion of the assays, data were available for all of the biomarker assays for 153 participants. Upon merging the biomarker assay data and MRI data, complete data (both complete biomarker and MRI data) were available for analysis in 151 participants. These participants were similar in age and sex distribution to those from the larger study sample.

The institutional review boards of Boston University Medical Center and the Veterans Administration Boston Health Care System approved the baseline and follow-up examinations. Informed consent was obtained from all study participants.

### Framingham sample

The Framingham study cohort consisted of two separate groups as follows: members of the Framingham Heart Study Offspring cohort, and a newly recruited cohort from the town of Framingham (MA, USA). Participants in this combined group, designated the 'Framingham Osteoarthritis Study cohort', were examined in 2002 to 2005.

Participants in the Framingham Heart Study Offspring cohort included surviving descendants (and spouses of descendants) of the original Framingham Heart Study cohort [25]. As part of a study of the inheritance of OA, selected participants in the Offspring cohort were originally examined between 1992 and 1994. Members of this group were identified as potential participants in the present study. All were contacted by telephone and invited to participate in the study. A validated survey instrument [26] supplemented by questions about medication use reflecting treated rheumatoid arthritis was used to exclude patients with rheumatoid arthritis.

The newly recruited cohort participants were drawn from a random sample from the Framingham, Massachusetts community. Flyers were hung in public areas to increase awareness of the study, which was focused on health, including bone health, foot health, and arthritis. Participants were recruited using random-digit dialing and census tract data to ensure that a representative sample of the Framingham community was recruited. To be included, individuals had to be at least 50 years old and ambulatory (use of assistive devices such as canes and walkers was allowed). Exclusion criteria were the presence of bilateral total knee replacements and the presence of rheumatoid arthritis. In neither Framingham group was participant selection based on the presence or absence of knee OA. The institutional review board of Boston University Medical Center approved the examinations.

Fasting blood and urine specimens were also obtained at the visit. For all participants biologic specimens were available, within 2 days, serum was frozen at -70°C and urine was frozen at -20°C. They were both then stored centrally at -70°C at the Biomedical Research Institute in Rockville (MD, USA).

After completion of the assays, data were available for all of the biomarker assays in 150 participants. Upon merging the biomarker assay data and MRI data, complete data (both complete biomarker and MRI data) were available for analysis in 144. These participants were similar to those from the larger study sample.

### Measures taken in both studies

Age, sex, and body mass index (BMI) were assessed in all study participants. BMI was calculated, using Quetelet's index, as weight (in kilograms) divided by height (in meters) squared. Height was measured using a stadiometer, and weight was measured using a balance beam scale.

### Magnetic resonance imaging

All subjects underwent a sagittal and a coronal T2 turbo-spin echo with fat suppression. The magnetic resonance system and pulse sequences for BOKS [22] and Framingham [27] have previously been described. Two different measures were used to define BMLs; a semiquantitative score and a quantitative BML volume.

BMLs were defined as ill-defined areas adjacent to the subarticular bone of hypointense signal on T_1_-weighted images and hyperintense signal on T_2_-weighted fat-suppressed images. Using the whole-organ MRI score system (WORMS) for sagittal images, each lesion was graded on a scale from 0 to 3, based on the extent of regional involvement [28]. BMLs were graded in each of 14 articular surface regions. The readers who performed all of the readings were blinded to the patients' characteristics and biomarker readings. Interobserver agreement for WORMS BML scores was good (intraclass correlation coefficients 0.69 to 0.88).

In addition, a subset of persons with BMLs underwent quantitative volume measurement of their BMLs using a proprietary software method (VirtualScopics, Rochester, NY, USA). BML volume (ml) was characterized using a BML segmentation multi-spectral match filter method. For the analyses presented here the BML volume refers to the total BML volume for the whole knee.

### Bone density measurement

In addition, the Framingham participants underwent measurement taken of bone mineral density (BMD). Because bone density may influence the levels of bone biomarkers and BMLs, we chose to use this as a covariate in our analyses. In our study, the data available for assessment of BMD were values for the femoral neck and lumbar spine. Because the interpretation of spine BMD can be complicated by the presence of compression fractures and degenerative disease, we chose femoral neck BMD as the better measure of systemic BMD. Measurements (in g/cm^2^) were obtained using dual-energy X-ray absorptiometry (Lunar Prodigy scanner; GE Lunar, Madison, WI, USA). The right femur was scanned unless there was a history of a previous fracture or joint replacement, in which case the left femur was scanned. We used standard positioning, as recommended by the manufacturer. For quality assurance, we scanned phantoms provided by the manufacturer under varying thicknesses of oil and water. No drift in BMD measurements was observed over time. We analyzed the proximal femur using the manufacturer's standard protocol, focusing on the femoral neck region for these analyses.

### Bone biomarkers

Biochemical markers of bone turnover were assayed in archived serum and urine stored at -70°C. We measured two serum bone formation markers: osteocalcin and bone-specific alkaline phosphatase (BSAP).

BSAP was measured using a monoclonal antibody that exhibits specificity for BSAP (Alkphase-B; Metra Biosystems, Mountain View, CA, USA), thus providing a quantitative measure of BSAP activity in serum as a measure of osteoblastic activity. The sensitivity is 0.7 U/l, and the intra-assay and inter-assay coefficients of variation at 28 U/l were 3.3% and 7.9%, respectively [29].

For osteocalcin, we used the US Food and Drug Administration approved Nordic Bioscience N-MID assay instead of an assay for intact osteocalcin. The reason for this is that circulating osteocalcin is unstable because it contains a tryptic cleavage site at amino acids 43 to 45 near to the carboxyl-terminus [30]. After cleavage, a large fragment containing amino acids 1 to 43 is formed, which is termed N-MID because it contains the amino (N)-terminus and middle portion of the protein. Whereas the levels of intact osteocalcin rapidly decreases, the total pool of intact osteocalcin and N-MID fragments remains stable in serum, even after repeated freeze thaw cycles or storage at elevated temperatures [31]. The N-MID osteocalcin enzyme-linked immunosorbent assay developed by Nordic Bioscience Diagnostics (Herlev, Denmark) measures the total pool of intact osteocalcin and N-MID fragments with equal affinity, ensuring reliable and robust measurements [31].

A bone resorption marker, type I collagen N-telopeptide (NTx) was measured in urine and corrected for urinary creatinine. Measurements of urinary NTx (nmol bone collagen equivalents/mmol creatinine) were performed by enzyme-linked immunosorbent assay (Osteomark NTx Urine; Ostex International, Seattle, WA, USA). The intra-assay coefficient of variation for this assy ranges from 5% to 19%. Urinary NTx values were normalized versus urinary creatinine to account for urinary dilution [32].

All assay measures were conducted at TNO (Leiden, The Netherlands). The assays were conducted blinded to outcomes.

### Analysis

Because of the effects of estrogen and bisphosphonates on bone remodeling markers, patients receiving these medications were removed from the analysis. Because of marked skewness, the natural log of each biomarker was used to predict BML. To facilitate comparison between the biomarkers, we used the standardized distribution of each biomarker as the predictor. We classified each participant according to maximum score of any BML (ordinal scale 0 to 3) and two binary outcomes: maximum score of BML > 0 and maximum score of BML > 1. We used contingency tables to assess levels of each biomarker in relation to status of BMLs. Logistic regression models were fit using BML as the outcome and biomarkers, age, sex, and BMI as predictors. A receiver operating characteristic curve was generated for each model, and the area under the curve was assessed. Finally, the biomarkers were used as predictors of quantitative BML volume in a linear regression.

The benefit of doing this analysis in Framingham was that it allowed us to adjust for the potential confounding influence of BMD. In addition, Framingham study had data on two knees; for this analysis a generalized estimating equation correction was used.

We also conducted exploratory analyses to assess whether bone biomarkers were associated with cartilage loss on MRI in the BOKS sample. We assessed whether increased levels of each biomarker was predictive of subsequent cartilage loss on knee MRI (ascertained from baseline to 30 month visit). We performed a logistic regression to examine the relation of level of NTx to the risk for cartilage loss in any plate. Cartilage loss was defined as an increase in cartilage score in any of the plates previously mentioned.

## Results

### BOKS analyses

A total of 151 individuals with knee OA were assessed (Table 1). The mean (standard deviation) age was 67 (9) years and 60% were male. Sixty-nine per cent of participants had a maximum BML score > 0 and 48% had a maximum BML score > 1.

**Table 1:** Characteristics of the BOKS study population

Parameter	Value
Age (years; mean ± SD [range])	67 ± 9 (50 to 93)
Sex (*n *[%] male)	91 (60%)
Proportion with BML > 0	69.5%
NTx/Cr (nM/mM; mean ± SD)	0.06 ± 0.12
BSAP (U/l; mean ± SD)	37.5 ± 23.2
Osteocalcin (U/l; mean ± SD)	19.2 ± 15.6

There were 151 participants. BOKS, Boston Osteoarthritis Knee Study; BSAP, bone-specific alkaline phosphatase; NTx/Cr, type I collagen N-telopeptide corrected for urinary creatinine; SD, standard deviation.

On investigation of the relation between biomarkers and semiquantitative BMLs, the only model that achieved statistical significance used maximum score of BML > 0 as the outcome. Results of this model are shown in Table 2. Although the overall model (including age, sex, and BMI) predicting BMLs was statistically significant for each biomarker, neither Ln-BSAP or Ln-osteocalcin exhibited a statistically significant univariate association with BMLs, regardless of how BMLs were defined. Ln-NTx exhibited a significant association with BMLs, with the odds of a BML being present increasing by 1.4-fold (95% confidence interval = 1.0-fold to 2.0-fold) per 1 standard deviation increase in the LnNTx, and with a small partial R^2 ^of 3.05. For Ln-BSAP, there was a modest nonsignificant association, with the odds of a BML being present increasing 1.4-fold per 1 standard deviation increase in the Ln-BSAP.

**Table 2:** BOKS analysis: independent association of standardized biomarkers with BML (BML > 0) per SD of change in marker

			Univariate association			Full model (including age, sex and BMI)	
Marker	(BML = 0)	(BML > 0)	OR	95% CI	R^2^	Full model R^2^	Full model ROC area

Ln-BSAP	3.48 (0.35)	3.57 (0.36)	1.4	(0. 9, 2.0)	2.58	14.56	0.701
Ln-osteocalcin	2.73 (0.51)	2.80 (0.55)	1.2	(0.8, 1.7)	0.8	13.41	0.687
Ln-NTx	-3.47 (1.07)	-3.27 (0.78)	1.4	(1.0, 2.0)	3.05	14.55	0.697

BMI, body mass index; BML, bone marrow; BOKS, Boston Osteoarthritis Knee Study; BSAP, bone-specific alkaline phosphatase; Ln, Natural Log; NTx, type I collagen N-telopeptide; SD, standard deviation.

A model that contained all three biomarkers together with age, sex, and BMI was also fit. This model had an R^2 ^value of 18.84%, which was significantly greater than the values for models with only one marker (likelihood ratio χ^2 ^= 21.1 on six degrees of freedom; *P *= 0.002). The odds ratios for this model were similar to those in the univariate models (odds ratio [95% confidence interval]): Ln-BSAP, 1.3 (0.9 to 2.0); Ln-osteocalcin, 1.2 (0.8 to 1.7); and Ln-NTx, 1.3 (0.9 to 2.0).

No significant relationship was found between biomarkers and quantitative BML volume in linear regression models in the 47 BOKS participants in whom BML volume was quantified. The exploratory analysis of standardized biomarkers of cartilage loss on MRI did not reveal any significant associations. The odds ratio (95% confidence interval) for Ln-NTx predicting cartilage loss after adjusting for age, sex, and BMI was 1.06 (0.76 to 1.48).

### Framingham analyses

A total of 144 participant in the Framingham Osteoarthritis Study were studied (Table 3). Their mean age was 68 years, their BMI was 29 kg/m^2^, and 40% of them were male. Fifty-five per cent of participants had a maximum BML score > 0.

**Table 3:** Characteristics of the Framingham study population

Age (years; mean ± SD [range])	68.5, 9.33 (49,90)
Body mass index (kg/m^2^; mean ± SD)	28.87 ± 5.24
Sex (% male)	39.9%
Proportion with BML > 0	54.9%
NTx/Cr (nM/mM; mean ± SD)	0.04 ± 0.02
BSAP (U/l; mean ± SD)	16.2 ± 7.5
Osteocalcin (U/l; mean ± SD)	33.9 ± 9.9

There were 144 participants. BSAP, bone-specific alkaline phosphatase; NTx/Cr, type I collagen N-telopeptide corrected for urinary creatinine; SD, standard deviation.

On investigation of the relation between biomarkers and semiquantitative BML, we did not find a significant relationship between BSAP and osteocalcin and BML > 0 (Table 4). The relationship between NTx and maximum score of BML > 0 revealed a significant association, with an odds ratio of 1.7 (95% confidence interval 1.1 to 2.6) after adjusting for age, sex, and BMI. After further adjusting for the presence of OA (Kellgren and Lawrence ≥ 2), the parameter estimates remained the same but the confidence limits were wider and the results no longer significant. On adding 'gender' as a predictor into the model there was no significant interaction, and so the sexes were combined. In the Framingham analysis, we had data for two knees, so generalized estimating equation was used; hence, there is no R^2 ^for this model.

**Table 4:** Framingham analysis: independent association of standardized biomarkers with BML (BML > 0) per SD of change in marker

Marker	Adjusted for age, sex, and BMI	Multi-adjusted (adjusted for age, sex, BMI, and BMD)	Multi-adjusted (Adjusted for OA, age, sex, BMI, and BMD)
Ln-BSAP	1.4 (0.9 to 2.1)	1.4 (0.9 to 2.0)	1.26 (0.75 to 2.11)
Ln-osteocalcin	1.1 (0.7 to 1.7)	1.1 (0.7 to 1.7)	1.60 (0.99 to 2.57)
Ln-NTx	1.7 (1.1 to 2.7)	1.7 (1.1 to 2.6)	1.65 (0.96 to 2.83)

Values are presented as odds ratio (95% confidence interval). BML, bone marrow; BSAP, bone-specific alkaline phosphatase; Ln, Natural Log; NTx, type I collagen N-telopeptide; SD, standard deviation.

On assessment of the relation between biomarkers and quantitative BML volume, no significant relationship was found between BSAP and quantitative BML volume in linear regression models (Table 5). In contrast, both osteocalcin and NTx exhibited a positive association (*P *< 0.04) after adjusting for femoral neck BMD.

**Table 5:** Framingham analysis: independent association of standardized biomarkers with BML volume

Marker	β Coefficient	*P *value	β Coefficient^a^	*P *value^a^
Ln-BSAP	0.07	0.67	0.16	0.33
Ln-osteocalcin	0.27	0.09	0.34	0.04
Ln-NTx	0.23	0.15	0.33	0.04

Sixty-nine participants were included in this analysis. ^a^Adjusted for femoral neck bone mineral density. BML, bone marrow; BSAP, bone-specific alkaline phosphatase; Ln, Natural Log; NTx, type I collagen N-telopeptide.

## Discussion

Serum NTx appears to be consistently weakly associated with the presence of BMLs. Based on the these results the effect of NTx is small and not clinically meaningful. Other individual bone biomarkers (osteocalcin and BSAP) do not appear to be consistently associated with the presence of BMLs.

Bone is a specialized connective tissue with an extensive extracellular matrix that has the unique ability to become calcified, thereby forming, in conjunction with cartilage, key components of the skeletal system. Its metabolism is characterized by two opposing processes: formation and resorption. Typically, diseases of bone are characterized by an alteration in the bone resorption/formation balance, and our initial hypothesis was that the presence of BMLs would be associated with a disturbance in bone turnover. This disturbance in turnover appears to be more consistent with increased resorption than formation, based upon our findings in the present study. This remains consistent with the notion that persons with knee OA are likely to have higher bone turnover than persons without OA based on other studies, and that BMLs may reflect a proxy for radiographic OA [18,33,34]. When we further investigated the sample of persons in Framingham to explore this further, there was almost complete concordance with persons who had a BML > 0 and persons with radiographic knee OA (K&L ≥ 2).

A number of cross-sectional studies have been conducted in OA that suggest that bone biochemical markers may be helpful in predicting progressive disease, although results are inconsistent. Garnero and coworkers [35] conducted a cross-sectional study of a group of 67 patients with knee OA (mean age 64 years, median disease duration 8 years) and 67 healthy control individuals. All bone turnover markers were decreased in patients with knee OA as compared with control individuals (-36%, -38%, and -52% [*P *< 0.0001] for serum osteocalcin, serum CTX-I, and urinary CTX-I, respectively).

It is important to note that these findings are not consistent with previous research, such as that showing the urinary excretion of pyridinium cross-links is significantly increased in patients with large joint OA and hand OA, suggesting an increased rate of bone turnover [34].

Recently, Garnero and colleagues [36] examined the relation between bone marrow abnormalities and CTX-II – a type II collagen biomarker that has been shown to predict radiographic progression in patients with knee OA [37]. Baseline CTX-II levels (but not serum CTX-I levels) were correlated (*r *= 0.29) with BMLs, and there was a suggestion that they predicted an increase in BML at 3 months.

Further longitudinal data also suggests that bone turnover is increased in OA. These data come from the population-based Chingford study, conducted in 216 women in whom urine from three time points was assayed for urinary collagen cross-link excretion (urine C-telopeptide of type I collagen (CTx) and NTx). Levels were found to be significantly elevated in knee OA patients as compared with age-matched control individuals without OA, and to the same increased levels as in women with osteoporosis. In addition, the group with progressive disease had higher levels than nonprogressors [33]. This study was done with conventional anteroposterior radiographs of the knee and evaluated overall changes in OA grade, and therefore the findings may not reflect cartilage loss. Thus, the highest quality studies suggest that bone turnover is elevated among those with OA, possibly a result of subchondral BMLs.

The results of previous studies of osteocalcin appear to be somewhat conflicting. A longitudinal study [38] showed that the 1-year increase in osteocalcin was associated with loss of joint space width in patients with knee OA over 3 years; however, the baseline measure did not predict progression of joint space narrowing. The longitudinal Michigan Bone Health Study [39] showed that serum osteocalcin was lower in 3-year incident cases of hand or knee OA as compared with women without OA.

Of the markers we studied, NTx appears to exhibit the greatest discrimination between participants with and those without BMLs. Type I collagen is predominant in the extracellular matrix of bone. Cross-linked N-telopeptides (NTx) have therefore been targeted as markers of bone resorption in urine. Elevated levels of urinary NTx indicate elevated human bone resorption [40], and our data suggest that their levels are increased in persons with BMLs. Based upon our findings, this investigative tool may assist in discriminating between persons with and without BMLs, but this discriminatory ability is weak at best.

There are some limitations of this work that warrant mention, some of which are generic to the application of biomarkers. Age-related increases are commonly seen in biochemical markers, and these may produce variation in both biomarker levels and BMLs [41]. Efforts were made to adjust for age in the analyses. The BOKS and Framingham studies assessed the local structural changes in participants' knees only. It may be that other studies that investigate the total body burden of OA, including other joint areas, may be able to detect an association in persons with symptomatic OA. Another potential explanation for our null findings in persons with symptomatic OA is that we have insufficient power. Similarly, bone biomarker assays may reflect alterations in bone metabolism that are present at the time the sample was collected, whereas the BML lesion seen on an MRI reflects a structural change that took place at some time in the past and may be stable, may progress, or may even regress. The markers chosen are not specific to OA but rather are likely to be altered in someone with rapidly remodelling bone, including fractures, bone forming tumours, and growing children. Furthermore, they reflect systemic sources, and the progression or lack thereof of OA [42], which is quite localized, may not contribute substantively to altered levels.

## Conclusion

Serum NTx was weakly associated with BMLs presence in both study samples. Changes in other markers were not associated consistently with the presence of BMLs. If local bone turnover in persons with symptomatic knee OA is associated with visible lesions, then they do not appear to affect systemic biomarker levels markedly. Based on these data, we would not advocate their use as markers for the presence of BMLs in persons with symptomatic knee OA.

## Abbreviations

BMD: bone mineral density; BMI: body mass index; BML: bone marrow lesion; BOKS: Boston Osteoarthritis Knee Study; BSAP: bone-specific alkaline phosphatase; Cr: creatinine; MRI: magnetic resonance imaging; NTx: type I collagen N-telopeptide; OA: osteoarthritis.

## Competing interests

The authors declare that they have no competing interests. The corresponding author had full access to all of the data used in the study and had final responsibility for the decision to submit for publication.

## Authors' contributions

DJH conceived of the study, participated in acquisition of the data, data analysis and interpretation, and manuscript preparation, and approved the final manuscript. ML participated in data analysis and interpretation, manuscript preparation, and approved the final manuscript. JL participated in data analysis and interpretation, and manuscript preparation, and approved the final manuscript. DCB participated in study design and manuscript preparation, and approved the final manuscript. MN participated in study design and manuscript preparation, and approved the final manuscript. JD participated in acquisition of the data and manuscript preparation, and approved the final manuscript. RP participated in study design and manuscript preparation, and approved the final manuscript. DE participated in study design and manuscript preparation, and approved the final manuscript. AG participated in acquisition of the data and manuscript preparation, and approved the final manuscript. DG participated in acquisition of the data and manuscript preparation, and approved the final manuscript. ST participated in acquisition of the data and manuscript preparation, and approved the final manuscript. DTF participated in study design, participated in acquisition of the data and manuscript preparation, and approved the final manuscript.

## References

[B1] Burr DB (2004). The importance of subchondral bone in the progression of osteoarthritis. J Rheumatol Suppl.

[B2] Patel V, Issever AS, Burghardt A, Laib A, Ries M, Majumdar S (2003). MicroCT evaluation of normal and osteoarthritic bone structure in human knee specimens. J Orthopaedic Res.

[B3] Burr DB (1998). The importance of subchondral bone in osteoarthrosis. Curr Opin Rheumatol.

[B4] Felson DT, Chaisson CE, Hill CL, Totterman SM, Gale ME, Skinner KM, Kazis L, Gale DR (2001). The association of bone marrow lesions with pain in knee osteoarthritis. Ann Intern Med.

[B5] Speer KP, Spritzer CE, Bassett FH, Feagin JA, Garrett WE (1992). Osseous injury associated with acute tears of the anterior cruciate ligament. Am J Sports Med.

[B6] McAlindon TE, Watt I, McCrae F, Goddard P, Dieppe PA (1991). Magnetic resonance imaging in osteoarthritis of the knee: correlation with radiographic and scintigraphic findings. Ann Rheum Dis.

[B7] Dieppe P, Cushnaghan J, Young P, Kirwan J (1993). Prediction of the progression of joint space narrowing in osteoarthritis of the knee by bone scintigraphy. Ann Rheum Dis.

[B8] Boegard T, Rudling O, Dahlstrom J, Dirksen H, Petersson IF, Jonsson K (1999). Bone scintigraphy in chronic knee pain: comparison with magnetic resonance imaging. Ann Rheum Dis.

[B9] Felson DT, McLaughlin S, Goggins J, LaValley M, Gale M, Totterman S, Li W, Hill C, Gale D (2003). Bone marrow edema and its relation to X-ray progression in knee osteoarthritis. Ann Intern Med.

[B10] Zanetti M, Bruder E, Romero J, Hodler J (2000). Bone marrow edema pattern in osteoarthritic knees: correlation between MR imaging and histologic findings. Radiology.

[B11] Mansell JP, Bailey AJ (1998). Abnormal cancellous bone collagen metabolism in osteoarthritis. J Clin Invest.

[B12] Mansell JP, Tarlton JF, Bailey AJ (1997). Biochemical evidence for altered subchondral bone collagen metabolism in osteoarthritis of the hip. Br J Rheumatol.

[B13] Karvonen RL, Miller PR, Nelson DA, Granda JL, Fernandez-Madrid F (1998). Periarticular osteoporosis in osteoarthritis of the knee. J Rheumatol.

[B14] Li B, Aspden RM (1997). Material properties of bone from the femoral neck and calcar femorale of patients with osteoporosis or osteoarthritis. Osteoporos Int.

[B15] Grynpas MD, Alpert B, Katz I, Lieberman I, Pritzker KP (1991). Subchondral bone in osteoarthritis. Calcif Tissue Int.

[B16] Hunter DJ, Spector TD (2003). The role of bone metabolism in osteoarthritis. Curr Rheumatol Rep.

[B17] Bettica P, Cline G, Hart D, Meyer J, Spector T (2002). Evidence for increased bone resorption in patients with progressive knee OA: longitudinal results from the Chingford study. Arthritis Rheum.

[B18] Hunter DJ, Hart D, Snieder H, Bettica P, Swaminathan R, Spector TD (2003). Evidence of altered bone turnover, vitamin D and calcium regulation with knee osteoarthritis in female twins. Rheumatology.

[B19] Dequeker J, Mohan S, Finkelman RD, Aerssens J, Baylink DJ (1993). Generalized osteoarthritis associated with increased insulin-like growth factor types I and II and transforming growth factor beta in cortical bone from the iliac crest. Possible mechanism of increased bone density and protection against osteoporosis. Arthritis Rheum.

[B20] Hart DJ, Cronin C, Daniels M, Worthy T, Doyle DV, Spector TD (2002). The relationship of bone density and fracture to incident and progressive radiographic osteoarthritis of the knee: the Chingford Study. Arthritis Rheum.

[B21] Zhang Y, Hannan MT, Chaisson CE, McAlindon TE, Evans SR, Aliabadi P, Levy D, Felson DT (2000). Bone mineral density and risk of incident and progressive radiographic knee osteoarthritis in women: the Framingham Study. J Rheumatol.

[B22] Felson DT, McLaughlin S, Goggins J, LaValley MP, Gale ME, Totterman S, Li W, Hill C, Gale D (2003). Bone marrow edema and its relation to progression of knee osteoarthritis. Ann Intern Med.

[B23] Altman R, Asch E, Bloch D, Bole G, Borenstein D, Brandt K, Christy W, Cooke TD, Greenwald R, Hochberg M (1986). Development of criteria for the classification and reporting of osteoarthritis. Classification of osteoarthritis of the knee. Diagnostic and Therapeutic Criteria Committee of the American Rheumatism Association. Arthritis Rheum.

[B24] Chaisson CE, Gale DR, Gale E, Kazis L, Skinner K, Felson DT (2000). Detecting radiographic knee osteoarthritis: what combination of views is optimal?. Rheumatology.

[B25] Felson DT, Naimark A, Anderson J, Kazis L, Castelli W, Meenan RF (1987). The prevalence of knee osteoarthritis in the elderly. The Framingham Osteoarthritis Study. Arthritis Rheum.

[B26] Karlson EW, Sanchez-Guerrero J, Wright EA, Lew RA, Daltroy LH, Katz J, Liang MH (1995). A connective tissue disease screening questionnaire for population studies. Ann Epidemiol.

[B27] Lo GH, Hunter DJ, Zhang Y, McLennan CE, LaValley MP, Kiel DP, McLean RR, Genant HK, Guermazi A, Felson DT (2005). Bone marrow lesions in the knee are associated with increased local bone density. Arthritis Rheum.

[B28] Peterfy CG, Guermazi A, Zaim S, Tirman PF, Miaux Y, White D, Kothari M, Lu Y, Fye K, Zhao S, Genant HK (2004). Whole-Organ Magnetic Resonance Imaging Score (WORMS) of the knee in osteoarthritis. Osteoarthritis Cartilage.

[B29] Gomez BJ, Ardakani S, Ju J, Jenkins D, Cerelli MJ, Daniloff G, Kung VT (1995). Monoclonal antibody assay for measuring bone-specific alkaline phosphatase activity in serum. Clin Chem.

[B30] Garnero P, Grimaux M, Seguin P, Delmas PD (1994). Characterization of immunoreactive forms of human osteocalcin generated *in vivo* and *in vitro*. J Bone Miner Res.

[B31] Rosenquist C, Qvist P, Bjarnason N, Christiansen C (1995). Measurement of a more stable region of osteocalcin in serum by ELISA with two monoclonal antibodies. Clin Chem.

[B32] Ju HS, Leung S, Brown B, Stringer MA, Leigh S, Scherrer C, Shepard K, Jenkins D, Knudsen J, Cannon R (1997). Comparison of analytical performance and biological variability of three bone resorption assays. Clin Chem.

[B33] Bettica P, Cline G, Hart DJ, Meyer J, Spector TD (2002). Evidence for increased bone resorption in patients with progressive knee osteoarthritis: longitudinal results from the Chingford study. Arthritis Rheumatism.

[B34] Stewart A, Black A, Robins SP, Reid DM (1999). Bone density and bone turnover in patients with osteoarthritis and osteoporosis. J Rheumatol.

[B35] Garnero P, Piperno M, Gineyts E, Christgau S, Delmas PD, Vignon E (2001). Cross sectional evaluation of biochemical markers of bone, cartilage, and synovial tissue metabolism in patients with knee osteoarthritis: relations with disease activity and joint damage. Ann Rheum Dis.

[B36] Garnero P, Peterfy C, Zaim S, Schoenharting M (2005). Bone marrow abnormalities on magnetic resonance imaging are associated with type II collagen degradation in knee osteoarthritis: a three-month longitudinal study. Arthritis Rheum.

[B37] Garnero P, Ayral X, Rousseau JC, Christgau S, Sandell LJ, Dougados M, Delmas PD (2002). Uncoupling of type II collagen synthesis and degradation predicts progression of joint damage in patients with knee osteoarthritis. Arthritis Rheum.

[B38] Bruyere O, Collette JH, Ethgen O, Rovati LC, Giacovelli G, Henrotin YE, Seidel L, Reginster JY (2003). Biochemical markers of bone and cartilage remodeling in prediction of longterm progression of knee osteoarthritis. J Rheumatol.

[B39] Sowers M, Lachance L, Jamadar D, Hochberg MC, Hollis B, Crutchfield M, Jannausch ML (1999). The associations of bone mineral density and bone turnover markers with osteoarthritis of the hand and knee in pre- and perimenopausal women. Arthritis Rheum.

[B40] Rosen HN, Dresner-Pollak R, Moses AC, Rosenblatt M, Zeind AJ, Clemens JD, Greenspan SL (1994). Specificity of urinary excretion of cross-linked N-telopeptides of type I collagen as a marker of bone turnover. Calcif Tissue Int.

[B41] Poole AR, Witter J, Roberts N, Piccolo F, Brandt R, Paquin J, Baron M (1990). Inflammation and cartilage metabolism in rheumatoid arthritis. Studies of the blood markers hyaluronic acid, orosomucoid, and keratan sulfate. Arthritis Rheum.

[B42] Hunter DJ, Li J, LaValley M, Bauer DC, Nevitt M, DeGroot J, Poole R, Eyre D, Guermazi A, Gale D, Felson DT (2007). Cartilage markers and their association with cartilage loss on magnetic resonance imaging in knee osteoarthritis: the Boston Osteoarthritis Knee Study. Arthritis Res Ther.

